# Mitochondrial E3 ligase MARCH5 is a safeguard against DNA-PKcs-mediated immune signaling in mitochondria-damaged cells

**DOI:** 10.1038/s41419-023-06315-9

**Published:** 2023-12-01

**Authors:** June Heo, Yeon-Ji Park, Yonghyeon Kim, Ho-Soo Lee, Jeongah Kim, Soon-Hwan Kwon, Myeong-Gyun Kang, Hyun-Woo Rhee, Woong Sun, Jae-Ho Lee, Hyeseong Cho

**Affiliations:** 1 Department of Biochemistry and Molecular Biology, Ajou University School of Medicine, Suwon, South Korea; 2 Department of Biomedical Sciences, Graduate School of Ajou University, Suwon, South Korea; 3 Department of Anatomy, College of medicine, Korea University, Seoul, South Korea; 4 Department of Infectious Diseases, Research Center of Infectious and Environmental Diseases, Armed Forces Medical Research Institute, Daejeon, South Korea; 5 Department of Chemistry, Seoul National University, Seoul, South Korea

**Keywords:** Pattern recognition receptors, Ubiquitylation

## Abstract

Mitochondrial dysfunction is important in various chronic degenerative disorders, and aberrant immune responses elicited by cytoplasmic mitochondrial DNA (mtDNA) may be related. Here, we developed mtDNA-targeted MTERF1-FokI and TFAM-FokI endonuclease systems to induce mitochondrial DNA double-strand breaks (mtDSBs). In these cells, the mtDNA copy number was significantly reduced upon mtDSB induction. Interestingly, in *cGAS* knockout cells, synthesis of interferon β1 and interferon-stimulated gene was increased upon mtDSB induction. We found that mtDSBs activated DNA-PKcs and HSPA8 in a VDAC1-dependent manner. Importantly, the mitochondrial E3 ligase MARCH5 bound active DNA-PKcs in cells with mtDSBs and reduced the type І interferon response through the degradation of DNA-PKcs. Likewise, mitochondrial damage caused by LPS treatment in RAW264.7 macrophage cells increased phospho-HSPA8 levels and the synthesis of *mIFNB1* mRNA in a DNA-PKcs-dependent manner. Accordingly, in *March5* knockout macrophages, phospho-HSPA8 levels and the synthesis of *mIFNB1* mRNA were prolonged after LPS stimulation. Together, cytoplasmic mtDNA elicits a cellular immune response through DNA-PKcs, and mitochondrial MARCH5 may be a safeguard to prevent persistent inflammatory reactions.

## Introduction

Mitochondrial DNA (mtDNA) is an independent genome with replication and transcription systems separate from nuclear DNA [1]. DNA insults originating from replication errors, ionizing radiation, and various chemicals affect both the mitochondrial and nuclear genomes, but their responses to damage are different [2]. The nucleus retains a variety of DNA damage response signaling and repair pathways tailored to different genotoxic stresses. Unlike the nucleus, when mitochondrial genomes encounter DNA double-strand breaks, mtDNA is primarily degraded by exonucleases of the mitochondrial replication machinery [3–5]. In these cases, a portion of the damaged and oxidized mtDNA molecules are released into the cytoplasm [6–8] through a mitochondrial permeability transition pore (mPTP) [9] and VDAC or BAX/BAK macropore channels [10, 11]. Mitochondrial RNAs (mtRNAs) are also released when mitochondrial herniation occurs [12, 13]. Aberrant release of mitochondrial nucleic acids (mtDNA and mtRNA) from damaged cells and tissues triggers the inflammatory response and immune response through different signaling pathways. Persistent immune signals are deleterious and may drive the host immunopathology of autoimmune and inflammatory disorders [8, 14–16], as well as various chronic degenerative disorders [17–19].

Intracellular DNA are sensed by pattern recognition receptors upon microbial infection or cellular damage. Among pattern recognition receptors [20, 21], the most recognized DNA sensor is cyclic GMP-AMP synthase (cGAS), which binds cytoplasmic double-stranded DNA (dsDNA) [22, 23]. This leads to the synthesis of cGAMP and the activation of stimulator of interferon genes (STING). The cGAS-STING pathway results in the activation of interferon regulatory factor 3 (IRF3) and nuclear factor kappa B, in turn promoting the transcription of type І interferon (IFN-I) and proinflammatory cytokines [24]. In addition, Toll-like receptor 9 and absent in melanoma 2 (AIM2) are two other major DNA-sensing receptors. Toll-like receptor 9 activates nuclear factor kappa B and IRF7, leading to the expression of genes encoding proinflammatory cytokines and interferons [25]. AIM2 also binds to dsDNA, leading to the formation of the AIM2 inflammasome that results in the proteolytic cleavage of IL-1β and IL-18 through caspase 1 activation [26, 27]. Recent reports also proposed DNA-PK as a cytoplasmic DNA sensor through a STING-independent DNA sensing pathway in which heat shock protein A8 (HSPA8/HSC70) is phosphorylated as a downstream factor [28]. To date, the cGAS-STING pathway is known to be a major driver of IFN-I response upon to cytoplasmic mtDNAs [6, 10]. However, it is possible that other DNA-sensing pathways associated with cytoplasmic mtDNA exist.

A mitochondrial MARCH5 E3 ubiquitin ligase belongs to the membrane-associated RING-CH (MARCH) family with 11 cellular homologs [29, 30]. MARCH5 is an important regulator of mitochondrial dynamics [31–33] and mitochondrial quality control by regulating the turnover of target proteins, which contributes to the maintenance of mitochondrial homeostasis [34–36]. Meanwhile, Viral E3 ligases K3 and K5, structurally similar to mammalian MARCH family, have immunosuppressive functions by regulating the expression of major histocompatibility complex І on the membrane surface of antigen-presenting cells [30]. Other cellular MARCH proteins also catalyze polyubiquitination of various immune receptors [37] or organelle membrane-associated components involved in innate immune responses. MARCH5 turns off persistent immune signaling by degrading oligomeric complexes of retinoic acid-inducible gene I (RIG-I) and mitochondrial antiviral-signaling protein (MAVS) formed upon RNA virus infection [38, 39]. Thus, MARCH5 on the mitochondria is emerged as important regulator of immune response.

In the present study, we hypothesized that the molecular system maintaining immune balance must be developed in cells with mtDNA damage to prevent immunopathogenic effects. To address this issue, we developed novel mtDNA-targeted MTERF1-FokI and TFAM-FokI systems that specifically generate mitochondrial DNA double-strand breaks (mtDSBs). We found that mtDSBs activated DNA-dependent protein kinase, catalytic subunit (DNA-PKcs) and HSPA8 in a cGAS/STING-independent manner. MARCH5 bound and degraded the activated DNA-PKcs, which contributes to switching off the IFN-I response. Thus, MARCH5 may be a safeguard to prevent persistent immune response.

## Results

### Specific targeting of mitochondrial DNA by the FokI endonuclease reduces the amount of mitochondrial DNA

**Fig. 1:**
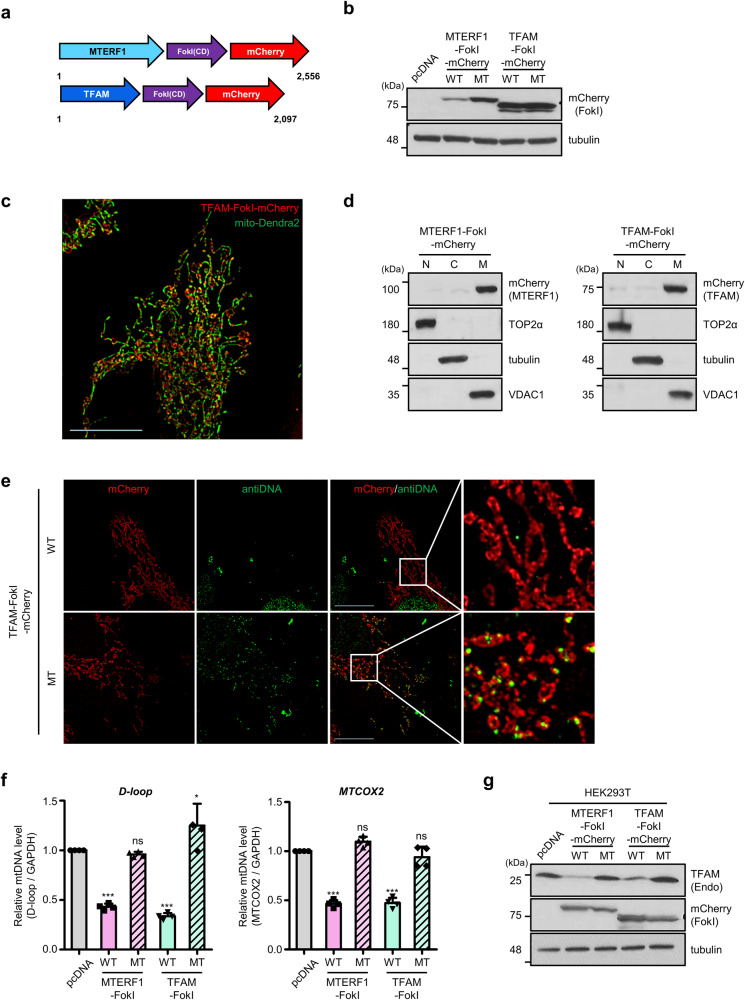
Specific targeting of mitochondrial DNA by the FokI endonuclease reduces the amount of mitochondrial DNA. **a** Schematic representation of the domain structure of the mitochondrial DNA-targeting FokI endonuclease. **b** Western blot analysis to confirm the expression of the indicated mt-FokI in the HeLa cell line harvested 36 h after transfection. WT, wild type, active mt-FokI; MT, catalytically inactive point mutant. **c** Representative immunofluorescence images visualizing the mitochondrial localization of TFAM-FokI in HeLa cell lines stably expressing mito-Dendra2 fluorescent protein using structured illumination microscopy. Scale bars, 10 μm. **d** Western blot analysis of mt-FokIs in nuclear, cytosolic, and mitochondrial fractions isolated from HeLa cell line transfected with the indicated mt-FokI. N, nuclear; C, cytosolic; M, mitochondrial fraction. **e** Immunofluorescence staining with anti-DNA antibody in HeLa cell line transfected with TFAM-FokI. Representative images with expressed TFAM-FokI in red and stained dsDNA in green. Scale bars, 10 μm. **f** Quantitative PCR (qPCR) for relative mtDNA copy number using primers in the *D-loop*, *MTCOX2* sequence in the HEK293T cell line, 36 h after indicated mt-FokI expression. Normalized expression Data are means ± SD of *n* = 4. Statistics were performed using one-way analysis of variance (ANOVA) with confidence interval = 95% and Bonferroni’s post hoc test. **g** Western blot analysis of endogenous TFAM in the same samples as shown in (**f**). Each experiment was performed at least three times. *P ≤ 0.05, ***P ≤ 0.005, ns, not significant.

To improve our understanding of how cells respond to cytoplasmic mtDNAs following mtDSBs, we created fusion genes by linking mtDNA binding proteins of MTERF1 (mitochondrial transcription termination Factor 1) (MTERF1-FokI) or TFAM (mitochondrial transcription Factor A) (TFAM-FokI) to the FokI endonuclease cleavage domain [40] and mCherry fluorescent protein (Fig. 1a). MTERF1 and TFAM bind specific regions of mtDNA [41–43] and TFAM also broadly binds to mtDNA in mitochondrial nucleoids [44, 45]. As a negative control, we used the catalytically inactive FokI endonuclease in which Asp454 of FokI is substituted with Ala (FokI MT). Both MTERF1-FokI-mCherry and TFAM-FokI-mCherry constructs (called these mt-FokIs, mtDNA-specific FokIs) were properly expressed in HeLa cells (Fig. 1b). The subcellular localization of these proteins was traced in mito-Dendra2 HeLa cells expressing the mitochondria-targeting Dendra2 fluorescent protein, and the expression of both TFAM-FokI and MTERF1-FokI colocalized with mitochondria under structured illumination microscopy and fluorescence microscopy (Fig. 1c and Supplementary Fig. 1a). This was verified by a cellular fractionation assay, which showed that most MTERF1-FokI and TFAM-FokI were found in the mitochondrial fraction (Fig. 1d).

We investigated the effect of mt-FokIs on mtDNA. In control HeLa cells, anti-DNA antibody [46] showed an extranuclear signal that was colocalized with mitochondria (Supplementary Fig. 1b). However, upon expression of TFAM-FokI WT or MTERF1-FokI WT, mtDNA staining was markedly decreased in both HeLa cells and hTERT-immortalized retinal pigment epithelial (hTERT-RPE1) cells. In contrast, decrease in mtDNA staining was not observed in cells expressing mutant mt-FokIs (Fig. 1e and Supplementary Fig. 1c). This was verified by determining mtDNA copy numbers. Relative mtDNA copy numbers were decreased by ~50% upon the expression of mt-FokI WTs but not mt-FokI MTs. (Fig. 1f and Supplementary Fig. 1d). Based on these results, we concluded that the expression of mt-FokI WTs induced mtDSBs and a subsequent decrease in mtDNA content. Notably, endogenous TFAM levels were downregulated in cells transfected with mt-FokIs (Fig. 1g), which is a common outcome of mtDNA damage [47, 48]. We determined whether mtDSBs alter mitochondrial parameters such as mitochondrial morphology and mitochondrial membrane potential. After transfection of TFAM-FokI WT and MT constructs into mito-Dendra2 HeLa cells, the mitochondrial particle size and number per cell were quantified. We found that average size of mitochondrial particles per cell was smaller in TFAM-FokI WT expressing cells than in FokI mutant cells. Meanwhile, the mitochondrial particle count per cell was higher in cells expressing TFAM-FokI WT (Supplementary Fig. 2a), suggesting that mitochondrial fragmentation occurs upon mtDSBs induction. Mitochondrial membrane potential was determined by staining the mitochondria with TMRM 24 h after transfection. We found that mitochondrial membrane potential did not differ between TFAM-FokI WT and MT expressing cells (Supplementary Fig. 2b). Collectively, we successfully devised fusion genes that induce DSBs specifically in mtDNA, which led to a significant decrease in the copy number of mtDNA.

### MtDSBs induce the type I interferon response in a cGAS-independent manner

**Fig. 2:**
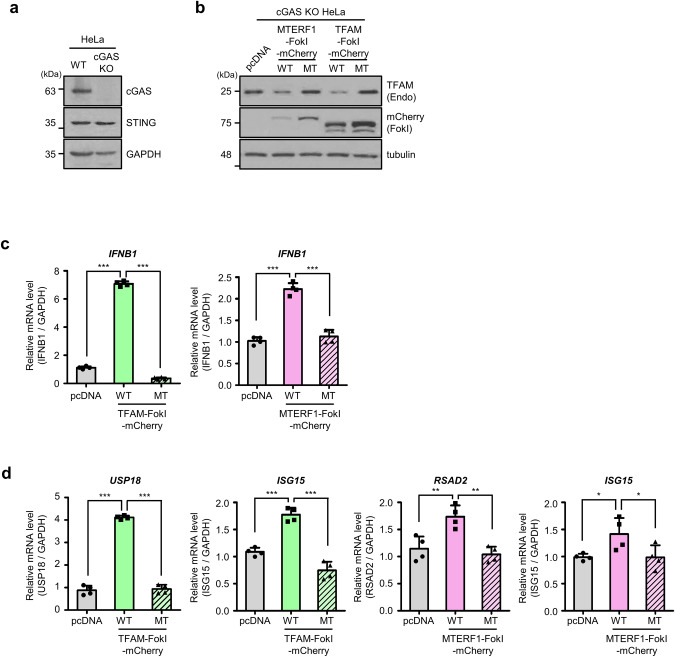
MtDSBs induce the type I interferon response in a cGAS-independent manner. **a** Western blot analysis to compare basal levels of cGAS and STING in WT and *cGAS* KO HeLa cell lines. WT, wild type; KO, knockout. **b** Western blot analysis of TFAM levels in *cGAS* KO HeLa cell lines harvested 36 h after transfection with the indicated mt-FokI. **c** RT-qPCR analysis of *IFNB1* mRNA in *cGAS* KO HeLa cells transfected with indicated TFAM- and MTERF1-FokI and collected 36 h after transfection. Mean ± SD of *n* = 4; one-way ANOVA with CI = 95% and Bonferroni’s post hoc test. **d** RT-qPCR analysis measuring mRNA of the indicated ISG after transfection of the indicated mt-FokI into *cGAS* KO HeLa cell lines. Data are means ± SD for *n* = 4 are shown. Each experiment was performed at least three times. *P ≤ 0.05, **P ≤ 0.01, ***P ≤ 0.005.

It has been widely accepted that damaged mtDNA is translocated to the cytoplasm that is monitored by the cGAS/STING signaling pathway, triggering the IFN-I response. Because an increasing number of cytoplasmic DNA sensing pathways are being identified [21], we attempted to examine whether alternative pathways exist to sense cytoplasmic mtDNAs in our mitochondria-specific DSBs model. To do that, we utilized *cGAS* knockout (KO) HeLa cells (Fig. 2a) and determined expression levels after transfection of MTERF1-FokI and TFAM-FokI constructs in these cells. Consistent with the results shown in Fig. 1g, endogenous TFAM levels were lower in cells transfected with MTERF1-FokI WT or TFAM-FokI WT than in cells transfected with MTERF1-FokI MT or TFAM-FokI MT (Fig. 2b), suggesting that mtDNA damage occurred. We then examined the IFN-I response by determining the mRNA levels of *IFNB1* and interferon-stimulated genes (ISGs). Interestingly, we observed that both TFAM-FokI WT and MTERF1-FokI WT increased the levels of *IFNB1* mRNA, whereas the catalytically dead forms of mt-FokIs did not (Fig. 2c). Similarly, the expression levels of ISG mRNA were also elevated by transfection of wild-type mt-FokIs, although elevated levels varied among different ISGs (Fig. 2d). In contrast, the levels of ISG mRNA in cells transfected with catalytically inactive mutant forms of mt-FokIs were similar to those in control cells transfected with pcDNA. Thus, the data suggest that mtDSBs induce the IFN-I response in a cGAS-independent manner.

### MtDSBs activate the DNA-PKcs-mediated IFN-I response

**Fig. 3:**
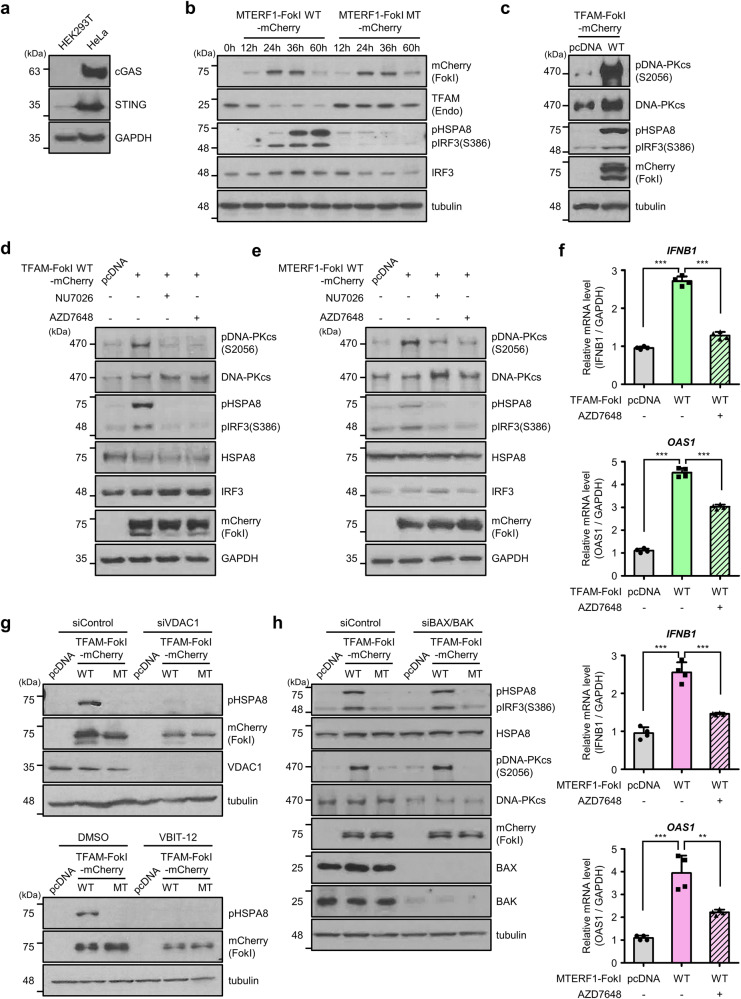
MtDSBs activate the DNA-PKcs-mediated type I interferon response. **a** Western blot analysis of endogenous cGAS and STING levels in HEK293T and HeLa cells. **b** Western blot analysis to determine the levels of pIRF3 and pHSPA8 in HEK293T harvested after expression of MTERF1-FokI WT or MT for the indicated times. **c** Western blot analysis of pDNA-PKcs, pHSPA8 and pIRF3 levels in lysates from HEK293T cells expressing TFAM-FokI WT for 36 h. **d**, **e** Western blot analysis of pDNA-PKcs, pHSPA8 and pIRF3 levels after expression of TFAM-FokI WT or MTERF1-FokI WT in HEK293T cells pretreated with 12.5 μM NU7026 and 3 μM AZD7648. **f** Analysis of *IFNB1*, *OAS1* mRNA levels in *cGAS* KO HeLa cells expressing TFAM-FokI WT or MTERF1-FokI WT pretreated with or without AZD7648. Data are means ± SD of *n* = 4; one-way ANOVA with CI = 95% and Bonferroni’s post hoc test. **g** Up, Western blot to analyze the levels of pHSPA8 after transfection of indicated TFAM-FokI into HEK293T cells with VDAC knockdown by siRNA. Down, Western blot analysis of pHSPA8 in cells collected 36 h after expression of the indicated TFAM-FokI in HEK293T cells pretreated with 80 μM VBIT-12. **h** Western blot to analyze the levels of pHSPA8, pIRF3 and pDNA-PKcs 36 h after transfection with the indicated TFAM-FokI in HEK293T cells transfected with siBAX and siBAK. Each experiment was performed at least three times. **P ≤ 0.01, ***P ≤ 0.005.

IRF3 is a major transcription factor that binds the canonical interferon response element sequence in the promoter of IFN-β and IFN-α [49]. HEK293T cells do not express cGAS and express low levels of STING proteins [22] (Fig. 3a), and we examined the levels of phospho-IRF3 (pIRF3) after mt-FokIs transfection in these cells. Expression of MTERF1-FokI WT in HEK293T cells led to a time-dependent increase in pIRF3 levels for up to 60 h (Fig. 3b). In addition, we observed the presence of an additional strong band, the size of which was ~20 kDa higher than that of pIRF3. This additional band appeared to be the phospho-form of HSPA8, which has been found in the DNA-PKcs-mediated antiviral pathway [28]. Interestingly, both phospho-HSPA8 (pHSPA8) and pIRF3 levels were markedly upregulated in response to mtDNA cleavage in cells transfected with mt-FokI WTs. In contrast, they were barely seen in cells transfected with mt-FokI MTs (Fig. 3b, Supplementary Fig. 3a). The presence of pHSPA8 by mtDNA cleavage was also observed in hTERT-RPE1 and U-2 OS cell lines (Supplementary Fig. 3b, c). Because phosphorylation of HSPA8 and IRF3 can be mediated by DNA-PKcs [28], we examined whether DNA-PKcs is activated in our system by determining the phosphorylation of DNA-PKcs on Ser2056. A marked increase in phospho-DNA-PKcs (pDNA-PKcs) levels accompanied by pHSPA8 was observed in cells expressing TFAM-FokI WT (Fig. 3c) and MTERF1-FokI WT (Supplementary Fig. 3d). In addition, selective inhibitors of DNA-PKcs, NU7026 [50] and AZD7648 [51] abrogated the pHSPA8 and pIRF3 levels (Fig. 3d, e). We next addressed whether inhibitors of DNA-PKcs also suppressed the IFN-I response in these cells. Indeed, increased mRNA levels of *IFNB1* and ISG in *cGAS* KO HeLa cells expressing mt-FokI WTs were diminished by treatment with AZD7648 (Fig. 3f) and NU7026 (Supplementary Fig. 3e). Likewise, the mRNA levels of *IFNB1* were abolished in cells expressing TFAM-FokI WT after knockdown of DNA-PKcs using siRNA for DNA-PKcs (Supplementary Fig. 3f). To this end, we addressed whether inhibition of VDAC suppresses DNA-PKcs-mediated signaling, since damaged mtDNA is released into the cytoplasm through the VDAC protein [10]. Indeed, either knockdown of VDAC by siRNA or treatment with VBIT-12, a specific inhibitor of VDAC oligomerization, abolished the increase in pHSPA8 levels in cells expressing TFAM-FokI WT (Fig. 3g). In contrast, co-depletion of BAX and BAK by siRNAs did not alter the activation of DNA-PKcs and the pHSPA8 levels in our systems (Fig. 3h). In addition, neither depletion of cyclophilin D, a major component of the mPTP (Supplementary Fig. 4a) nor treatment with cyclosporin A, an inhibitor of mPTP (Supplementary Fig. 4b) did significantly affect them. Collectively, our data suggest that damaged mtDNA is released into the cytoplasm via VDAC and that the released mtDNA activates DNA-PKcs, leading to IFN-I response.

### Mitochondrial E3 ligase MARCH5 reduces the protein levels of DNA-PKcs and suppresses the type І IFN response

**Fig. 4:**
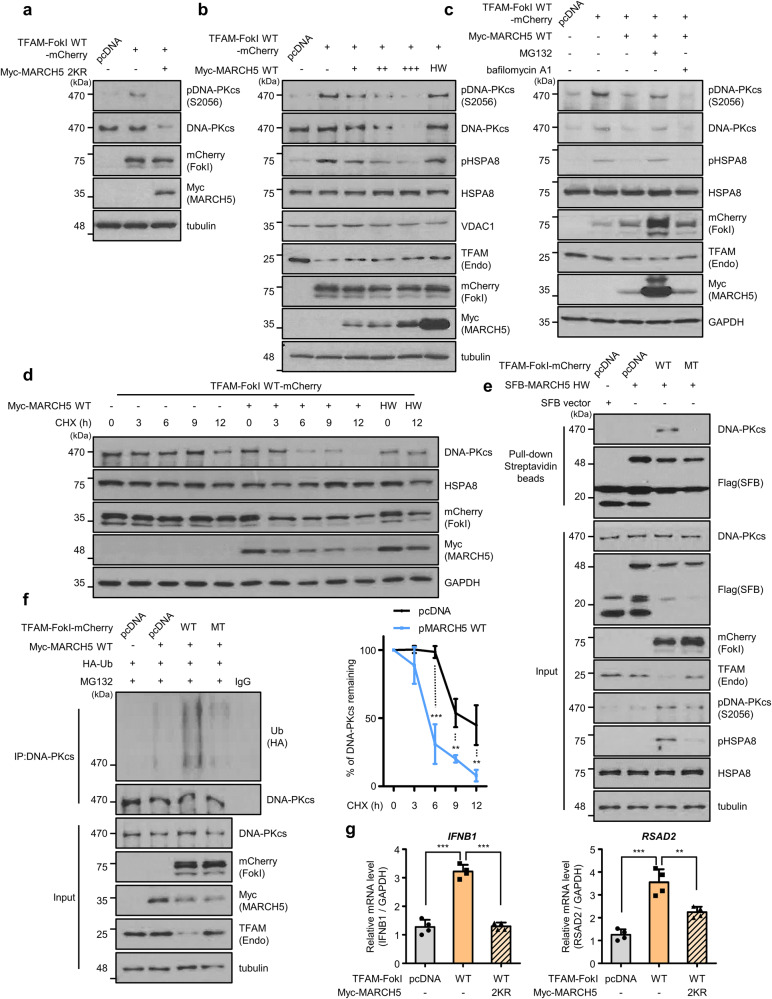
Mitochondrial E3 ligase MARCH5 reduces the protein levels of DNA-PKcs and suppresses the type І IFN response. **a** Western blot analysis of pDNA-PKcs and DNA-PKcs in HEK293T cells cotransfected TFAM-FokI WT with MARCH5 2KR for 36 h; 2KR, Point mutant with prevented self-ubiquitination of MARCH5. **b** Western blot analysis of DNA-PKcs, pHSPA8, HSPA8 and VDAC1 in HEK293T cells cotransfected with dose-dependent MARCH5 WT or single dose HW with TFAM-FokI WT for 36 h; HW, catalytically inactive point mutant of MARCH5. **c** Western blot analysis of DNA-PKcs and pHSPA8 in HEK293T cells cotransfected with TFAM-FokI WT and MARCH5 WT. Cells were untreated or treated with 10 μM proteasome inhibitor MG132 or 100 nM autophagosome-lysosome fusion inhibitor bafilomycin A1 12 h before harvest. **d** Western blot analysis of DNA-PKcs and HSPA8 levels with or without MARCH5 overexpression in HEK293T cells transfected with TFAM-FokI WT and treated with 15 μg/ml CHX for the indicated times. Graph, Normalized expression Data are means ± SD of *n* = 3 independent experiments; two-way ANOVA with CI = 95% and Bonferroni’s post hoc test. **e** Analysis of the interaction between DNA-PKcs and MARCH5 in HEK293T cells. Cells were cotransfected with the indicated TFAM-FokI and SFB-MARCH5 HW and harvested 36 h later. Cell lysates were immunoprecipitated with streptavidin beads and then immunoblotted with the antibodies against indicated proteins. **f** Ubiquitination assay of endogenous DNA-PKcs after immunoprecipitation with anti-DNA-PKcs antibody in HEK293T cells expressing the indicated TFAM-FokI and Myc-MARCH5 WT. Cells were treated with 10 μM MG132 12 h before harvest. Ubiquitination levels were measured using anti-Ub antibody. **g** RT-qPCR analysis of *IFNB1* and *RSAD2*, 36 h after cotransfection of TFAM-FokI WT and MARCH5 2KR in *cGAS* KO HeLa cells. Data are means ± SD of *n* = 4; one-way ANOVA with CI = 95% and Bonferroni’s post hoc test. Each experiment was performed at least three times. **P ≤ 0.01, ***P ≤ 0.005.

Uncontrolled immune responses elicited by accumulated cytoplasmic mtDNAs can be linked to various chronic inflammatory diseases [10, 19, 52] and thus, the cells may turn on molecular devices to control the immune balance. Mitochondrial-resident E3 ligases contribute to preserving cellular homeostasis by responding to various cellular stresses. They regulate mitochondrial dynamics, apoptosis, mitophagy and immune signaling [53–55]. Among these, the MARCH5 E3 ligase has the ability to target and degrade the activated RIG-I and MAVS complexes in innate immunity [38, 39], preventing the development of pathological progression. We therefore investigated whether MARCH5 also regulates the inflammatory response by targeting DNA-PKcs in our model. We expressed MARCH5 in cells transfected with TFAM-FokI WT and determined the protein levels of DNA-PKcs. We took advantage of MARCH5 2KR, in which two Lys residues at 40 and 54 are replaced by two Arg residues to prevent autoubiquitination of MARCH5 and its degradation [34]. Both MARCH5 2KR (Fig. 4a) and MARCH5 WT (Supplementary Fig. 5a) diminished the DNA-PKcs levels with a concomitant reduction in the pDNA-PKcs levels. Increasing concentrations of MARCH5 WT showed a dose-dependent decrease in both DNA-PKcs and pHSPA8 levels, while total HSPA8 levels remained unchanged (Fig. 4b). In contrast, MARCH5 HW [31] did not affect the protein levels of DNA-PKcs and pHSPA8. We also observed that MG132, a proteasome inhibitor, restored the reduced DNA-PKcs levels by MARCH5, whereas bafilomycin A1, a lysosome inhibitor, did not (Fig. 4c). In a cycloheximide (CHX) chase assay, the half-life of DNA-PKcs protein in response to mtDSBs was determined as 10–11 h. When MARCH5 WT was overexpressed in these cells, its half-life was diminished to 5-6 h. In contrast, the level of DNA-PKcs did not change in cells expressing E3 ligase-defective MARCH5 HW mutant (Fig. 4d). Inversely, when MARCH5 was depleted in MARCH5 KO cells [38], the half-life of DNA-PKcs was significantly extended (Supplementary Fig. 5b, c). Next, we cotransfected TFAM-FokI WT and SFB-MARCH5 HW (Fig. 4e) or SFB-MARCH5 2KR cotreated with MG132 (Supplementary Fig. 5d) to prevent the degradation of DNA-PKcs and performed a pull-down assay using streptavidin beads. Interestingly, both SFB-MARCH5 HW and 2KR interacted with endogenous DNA-PKcs following TFAM-FokI WT expression. In contrast, MARCH5 did not bind DNA-PKcs following TFAM-FokI MT expression (Fig. 4e, Supplementary Fig. 5d). In immunofluorescence staining, we found that pDNA-PKcs was concentrated on the mitochondria of the TFAM-FokI WT-expressing cells whereas its presence on the mitochondria was much less in the TFAM-FokI MT-expressing cell (Supplementary Fig. 5e). The data suggest that cytoplasmic DNA-PKcs moved to mitochondria upon induction of mtDSBs. MARCH5 binding to activated DNA-PKcs increased the ubiquitylation of DNA-PKcs, which did not occur in cells expressing TFAM-FokI MT (Fig. 4f). Consistently, introduction of MARCH5 2KR (Fig. 4g) or MARCH5 WT (Supplementary Fig. 5f) into TFAM-FokI WT-expressing cells reduced the mRNA levels of *IFNB1* and ISG (RSAD2). Together, these data indicate that MARCH5 binds and degrades activated DNA-PKcs in cells with mtDSBs and reduces the IFN-I response.

### MARCH5 prevents prolonged type І IFN signaling in murine macrophages stimulated by LPS

**Fig. 5:**
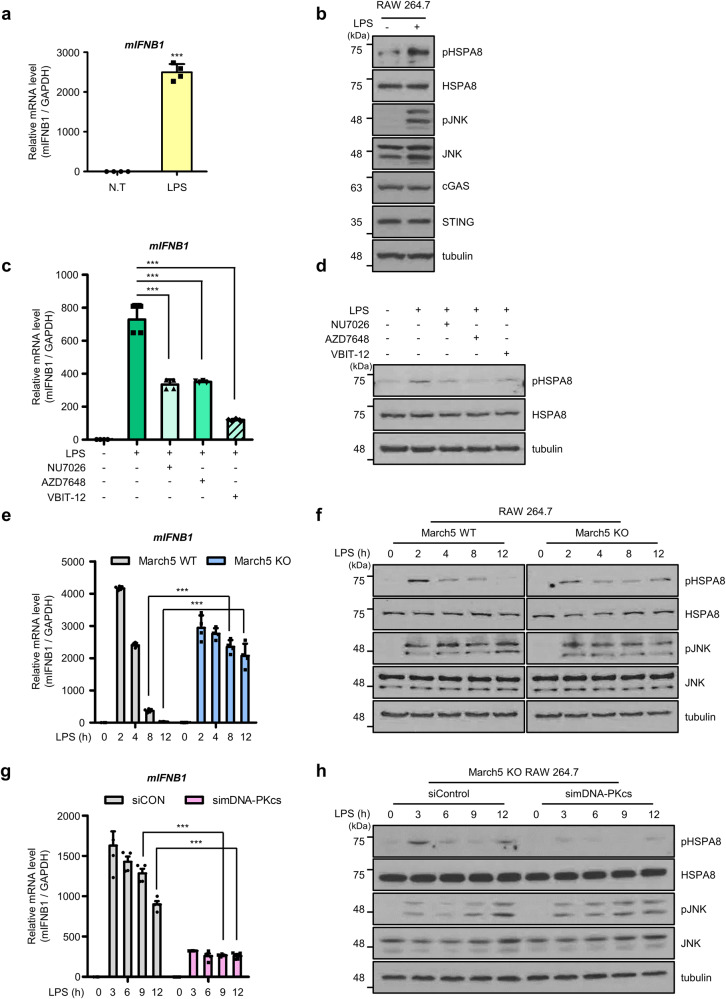
MARCH5 prevents prolonged type І IFN signaling in murine macrophages stimulated by LPS. **a** RT-qPCR analysis of *mIFNB1* mRNA levels in RAW 264.7 cells harvested after 4 h of treatment with 200 ng/ml LPS. Data are means ± SD of *n* = 4; one-tailed ratio unpaired Student’s *t*-test with CI = 95%, *P* values are shown at the top of the graph. **b** Experiment as in Fig. 6a. Western blot analysis of pHSPA8 and pJNK, cGAS and STING. **c**, RT-qPCR analysis of *mIFNB1* mRNA in RAW 264.7 cells pretreated with 12.5 μM NU7026, 3 μM AZD7648, and 80 μM VBIT-12 as indicated and treated with 200 ng/ml LPS. Data are means ± SD of *n* = 4; one-way ANOVA with CI = 95% and Bonferroni’s post hoc test. **d** Experiment as in Fig. 6c. Western blot analysis of pHSPA8. **e** RT-qPCR to measure *mIFNB1* mRNA levels after harvest of WT and *March5* KO RAW 264.7 cells treated with LPS for the indicated times. Normalized expression Data are means ± SD of *n* = 4 independent experiments; two-way ANOVA with CI = 95% and Bonferroni’s post hoc test. **f** Experiment as in Fig. 6e. Western blot analysis of pHSPA8 and pJNK. **g** RT-qPCR to measure m*IFNB1* mRNA levels after harvest of *March*5 KO RAW 264.7 cells transfected with si*mDNA-PKcs* for 72 h and treated with LPS for the indicated times. Normalized expression data are the mean ± SD of *n* = 4 independent experiments. Two-way analysis of variance with CI = 95% followed by Bonferroni post hoc test. ***h***, The cell lysates were prepared as described in 6g. Western blot analysis of pHSPA8 and pJNK. Each experiment was performed at least three times. ***P ≤ 0.005.

We next attempted to investigate the functional importance of the DNA-PKcs-mediated IFN-I response and MARCH5 under physiologically relevant conditions. Lipopolysaccharide (LPS) treatment stimulates the production of TNF-α and other cytokines and triggers inflammatory responses. It also causes mtDNA damage and ROS production [8, 56–58]. Since macrophages are the first line of defense against pathogens [59], we utilized the murine macrophage cell line RAW 264.7 and treated them with 200 ng/ml LPS for 2–12 h. We excluded the possibility of inflammasome pathways being involved in these cells because RAW 264.7 cells do not express ASC, an inflammasome adaptor protein [60]. LPS treatment increased the mRNA levels of *mNOS2* and *mCybb* [56] (Supplementary Fig. 6a). Upon LPS treatment for 4 h, a significant increase in *mIFNB1* mRNA levels was found (Fig. 5a). In addition, a substantial increase in phosphorylated mHSPA8 levels was accompanied by elevated phospho-JNK (pJNK) levels that were activated by TLR4 signaling (Fig. 5b). Unfortunately, the phosphorylated form of murine DNA-PKcs on Ser2053 was not detected by commercially available antibodies. To determine whether the induction of *mIFNB1* mRNA is dependent on the DNA-PKcs pathway, we treated cells with DNA-PKcs inhibitors and found that *mIFNB1* mRNA levels were diminished by more than 50%. In addition, treatment with a VDAC inhibitor significantly diminished *mIFNB1* mRNA levels by ~80% (Fig. 5c). Under these conditions, the increase in pHSPA8 levels induced by LPS treatment was also reduced by either the DNA-PKcs or the VDAC inhibitor treatment (Fig. 5d). Thus, the data suggest that DNA-PKcs contributes to the LPS-induced IFN-I response in murine macrophages. Next, to determine the role of MARCH5 in the LPS-induced IFN-I response, we generated *March5* KO RAW 264.7 cells by using the TALEN gene editing system [61]. The expression of *March5* mRNA levels in *March5* KO cells was significantly lower than that in *March5* WT macrophages (Supplementary Fig. 6b). When *March5* WT and KO cells were treated with LPS for 2–12 h, *mIFNB1* mRNA levels of *March5* WT cells were much lower than those in *March5* KO cells at 8–12 h (Fig. 5e), showing a significant difference in their levels at the same time points. Of note that *mIFNB1* mRNA levels in *March5* KO cells remained largely high after 8–12 h. In these experimental conditions, increased pJNK levels in both cell lines were maintained until 12 h (Fig. 5f) whereas pHSPA8 levels were different in these cells. *March5* WT macrophages showed a peak in pHSPA8 levels at 2 h after LPS treatment, which declined over time (Fig. 5f). However, the pHSPA8 levels in *March5* KO cells were mostly maintained for up to 12 h. Moreover, co-depletion of *March5* and *mDNA-PKcs* resulted in a profound reduction in *mIFNB1* mRNA, compared to those in *March5* KO cells (Fig. 5g, Supplementary Fig. 6c), Similarly, the pHSA8 level was barely detected after co-depletion of *mDNA-PKcs* and *March5* (Fig. 5h). Thus, these results suggest that *March5* modulates the DNA-PKcs-mediated IFN-I response upon LPS stimulation, thereby preventing prolonged immune signaling. Collectively, we suggest that MARCH5 acts as a safeguard molecule that regulates immune signaling dependent on the DNA-PKcs-HSPA8 axis.

## Discussion

**Fig. 6:**
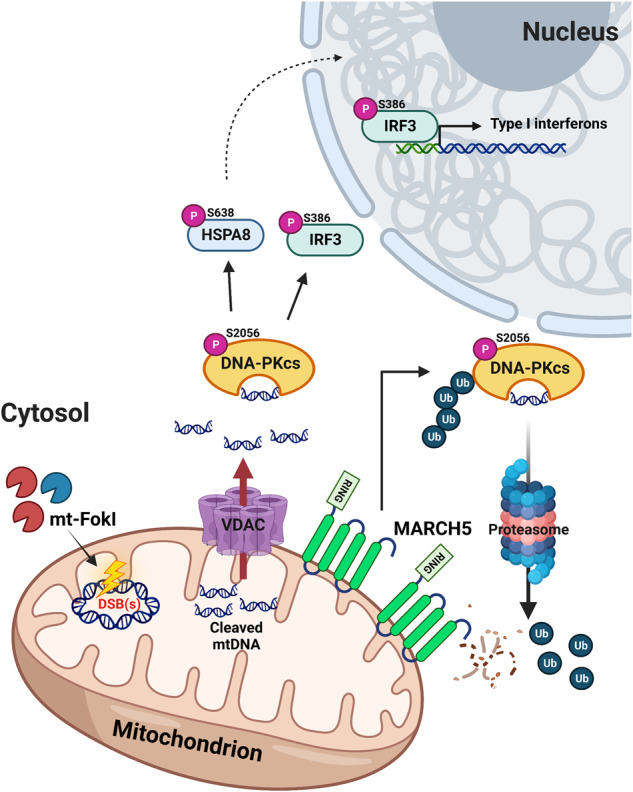
MARCH5 induces the degradation of DNA-PKcs activated by mtDSBs.

The instability of the mitochondrial genome and mtDNA insult can impair the function of mitochondria in various biological processes and lead to mitochondrial pathologies and inflammation-related degenerative diseases. In this study, we developed MTERF1-FokI and TFAM-FokI endonucleases capable of inducing DSBs only in mtDNA, thus establishing a system to study intracellular responses associated with mtDSBs. Using these mt-FokI systems, we observed an IFN-I response in cells with mtDSBs. We demonstrated that DNA-PKcs acts as a sensor to recognize cytoplasmic mtDNA derived from breaks formation in mtDNA. Furthermore, our data suggest that the mitochondrial E3 ligase MARCH5 regulates the DNA-PKcs-mediated immune signaling pathway and balances the immune response through ubiquitination and degradation of DNA-PKcs activated by mtDSBs induction or LPS treatment (Fig. 6), as shown in our model.

MTERF1 and TFAM were used as binding factors for mtDNA in our mt-FokI system. Both MTERF1 and TFAM are factors involved in mtDNA transcription and bind specifically to mtDNA, but they differ in the number of sites to which they bind [1]. MTERF1 is a single transcription termination factor that binds to the *MT-TL1* locus. In contrast, TFAM is a transcription initiation factor, and binds upstream of the transcription start site. There are at least three binding sites for TFAM to mtDNA, and the number of binding sites for TFAM on mtDNA increases further when considering sequence-independent binding to form nucleotide structures. Thus, the MTERF1-FokI and TFAM-FokI endonucleases are thought to generate mtDSBs at different frequencies. Upon mtDSBs induction, circular mtDNA is cleaved into one or two linear forms by MTERF1-FokI, whereas mtDNA may be cleaved into fragments by TFAM-FokI. Indeed, our data showed that both MTERF1-FokI and TFAM-FokI induced a severe reduction in mtDNA copy number (Fig. 1f). Thus, it appears that expression of mt-FokIs in cells leads to the degradation of mtDNA probably by the exonuclease activity of mitochondrial polymerase gamma and MGME1 after mtDNA resection [3, 4]. We also verified whether the cellular response in mt-FokI WT-expressing cells solely comes from mtDSBs. A previous report showed that TFAM also binds to nuclear DNA [62]. When HEK293T cells were transfected with mt-FokI WTs, there were few changes in the levels of γH2AX and pATM, whereas neocarzinostatin (NCS), a radiomimetic drug, substantially increased γH2AX and pATM levels (Supplementary Fig. 6d).

In a previous study [13], a mitochondrial-targeted transcription activator-like effector nuclease (TALEN) system similar to our mt-FokI system has been used to induce mtDSB in ARPE-19 cells that express low basal levels of cGAS, STING, and ZBP1. Interestingly, the authors showed that cytoplasmic double-stranded RNA sensor RIG-I is the major sensor that recognizes cytoplasmic mtRNA and induces the IFN-I response. In addition to cGAS, RIG-I, we here demonstrated that DNA-PKcs is another sensor of cytoplasmic mtDNAs in cells with mitochondrial damage. They also showed that BAX and BAK form oligomers in response to mtDSB, and contribute to the release of mitochondrial nucleic acids. Normally, BAX and BAK are activated under severe apoptotic conditions such as DNA damage, oxidative stress, or nutrient deprivation [11, 63]. In our system, depletion of VDAC1 and disruption of oligomerization reduced the phosphorylation of HSPA8 (Fig. 3g), indicating cleaved mtDNA in our system is released into the cytoplasm via VDAC. It is possible that VDAC is mainly responsible for the release of damaged mtDNAs when apoptotic signals are not induced.

In our experiments, the mtDSBs-mediated immune response may represent a chronic inflammatory response in which the secretion of IFN-β is mild but the stimulus to the cell remains prolonged. On the other hand, LPS treatment may represent an acute inflammatory response in which the cell is infected with a foreign pathogen and a burst of IFN-β is secreted. Notably, both acute and chronic inflammatory responses are initiated by mtDNA-mediated DNA-PKcs activation. Finally, we identified MARCH5 as a DNA-PKcs-mediated immune signaling brake. Recently, we found that MARCH5 regulates the activity of MAVS and RIG-I, two key proteins that play a role in the innate immune response to dsRNA virus infection, by inducing their degradation. MARCH5 interacts with oligomeric RIG-I, which binds to double-stranded RNA and undergoes a conformational change, and MAVS, which subsequently forms prion-like aggregates, but not with inactive RIG-I and MAVS [38, 39]. Thus, MARCH5 appears to have an increased binding affinity for proteins that have acquired insoluble properties, such as oligomers or aggregates. In this study, we showed that the interaction of MARCH5 with DNA-PKcs occurred only against to phosphorylated DNA-PKcs (Fig. 4e, Supplementary Fig. 5d). It is likely that various aspects of the DNA-PKcs structure are affected by phosphorylation, resulting in an increased binding affinity for MARCH5.

In conclusion, our results demonstrate that severe perturbations in mitochondrial genome integrity alert the nucleus through DNA-PKcs-mediated signaling and MARCH5 plays a vital role in maintaining cellular homeostasis by balancing the resulting excessive immune response. Understanding the role of MARCH5 in the inflammatory response may provide new insights into the regulatory mechanisms of several immune diseases and impact the development of new therapies for autoimmune and neurodegenerative diseases.

## Materials and methods

### Plasmids and chemicals

To generate mCherry-tagged FokI endonucleases targeting the mtDNA (MTERF1- and TFAM-FokI-mCherry), MTERF1, TFAM, the cleavage domain of FokI and mCherry were amplified from template DNA by PCR and cloned into pcDNA3.1(+) vector. Catalytically inactive mt-FokIs were generated by mutagenesis PCR from mt-FokI WTs using a site-directed mutagenesis kit (iNtRON biotechnology, Seongnam, South Korea). SFB-tagged MARCH5 was amplified by PCR and cloned into the SFB vector. Myc- or SFB-tagged MARCH5 mutants (MARCH5 H43W, MARCH5 2KR) were generated by mutagenesis PCR using Myc- or SFB-MARCH5 WT as a template. AZD7648 (#S8843), NU7026/LY293646 (#S2893), VBIT-12 (#S8936), Cycloheximide (CHX, #S7418), Cyclosporin A (CsA, #S2286) were purchased from Selleckchem (Houston, TX, USA) and dissolved to volume in Dimethyl sulfoxide (MERCK, Rahway, NJ, USA).

### Cell culture

HeLa ((ATCC, Manassas, VA, USA), CCL-2), HEK293T (ATCC, ACS-4500), RAW 264.7 (ATCC, TIB-71), and U-2 OS (ATCC, HTB-96) cells were cultured in DMEM-high (WELGENE, Gyeongsan, South Korea) supplemented with 10% heat-inactivated fetal bovine serum (FBS) (Gibco-BRL, Grand Island, NY, USA) and 1% penicillin/streptomycin (Gibco-BRL) in a 5% CO_2_ incubator at 37 °C. hTERT-RPE-1 (ATCC, CRL-4000) cells were cultured in DMEM/F-12 (Gibco-BRL) supplemented with 10% heat-inactivated fetal bovine serum (FBS) and 1% penicillin/streptomycin (Gibco-BRL) in a 5% CO_2_ incubator at 37 °C. The MycoFluor™ Mycoplasma Detection Kit (Thermo Fisher Scientific, Waltham, MA, USA) was used to confirm that all cell lines were free of mycoplasma contamination.

### Transfection and RNA interference

Plasmid DNA constructs were transfected using polyethylenimine (Polysciences, Warrington, PA, USA). Human-specific siRNA was custom-synthesized by BIONEER (Daejeon, South Korea). siRNAs were transfected using Lipofectamine 2000 (Thermo Fisher Scientific) according to the manufacturer’s instructions. A list of primers is provided in Supplementary Table 1.

### Generation of *March5* KO RAW264.7 and *cGAS* KO HeLa cell line

To generate *March5* KO RAW 264.7 cells, TALEN (transcription activator-like effector nuclease) plasmids [61] targeting exon 2 of *March5* were engineered by ToolGen (Seoul, South Korea). The MARCH5-specific TALEN plasmid contains the sequences of 5’-TGATGAAGATGATAGAACAG-3’ (TALEN-Left) and 5’-TCCTCTGCACCTGCATGGTC-3’ (TALEN-Right) that are linked by the EN target sequence. To enrich the *March5* KO cells, the pRG2S surrogate reporter plasmids [61] were cotransfected into RAW 264.7 cells using Lipofectamine 2000 according to the manufacturer’s protocol. The pRG2S reporters are composed of genes encoding two fluorescent proteins (red and green) and the frameshift mutations result in the restoration of the green fluorescent protein gene. At 48 h post-transfection, green fluorescent cells were sorted by FACS (FACS Vantage, BD Biosciences, Franklin Lakes, NJ, USA) and seeded at serial dilutions on 96-well plate to obtain a single cell colony. Two weeks later, cells were harvested to determine *March5* mRNA expression levels. *cGAS* KO HeLa cells were kind gifts from prof. Jae-Ho Lee (Ajou University School of Medicine, Suwon, South Korea).

### Western blot analysis

For Western blot analysis, cell lysates were lysed with RIPA buffer supplemented with protease and phosphatase inhibitors. Cell lysates were homogenized and centrifuged to remove cell debris. Protein concentrations were determined by Bradford assay, separated on 4–20% gradient SDS-PAGE, and transferred to the nitrocellulose membrane (GE Healthcare, Chicago, IL, USA). The Membrane was blocked with 5% bovine serum albumin (BSA) for 1 h at room temperature and incubated with primary antibodies overnight at 4 °C. The membrane was then incubated with peroxidase-linked secondary antibodies for 1 h at room temperature. Blots were visualized with an ECL system (GE Healthcare). A complete list of the antibodies used in the study and their relative dilutions is provided in Supplementary Table 2. Uncropped Western blot images can be found in the Supplemental Material.

### Mitochondrial fractionation

Mitochondria were isolated from HeLa cells. Cells were harvested in ice-cold PBS, washed, and suspended in medium A (250 mM sucrose, 2 mM HEPES (pH 7.4), 0.1 mM EGTA). Cells were incubated on ice for 10 min and then homogenized 5-6 times using a Dounce homogenizer. The homogenate was centrifuged at 571 × *g* for 20 min at 4 °C. The supernatant was collected and centrifuged again at 7000 × *g* for 10 min at 4 °C and separated into supernatant (cytosolic fraction) and pellet (mitochondrial fraction). The pellet was washed with 0.5 ml medium A, then resuspended in RIPA (supplemented with protease inhibitor) and sonicated. The lysate was centrifuged at 10,000 × *g* for 30 min at 4 °C. The supernatant was collected and transferred to a new tube. The absence of mitochondrial contamination in the supernatant was confirmed using an α-VDAC1 antibody.

### Cycloheximide chase assay

Cycloheximide (15 μg/ml) was added for indicated time periods to cells. Cells were lysed and equal amounts of proteins were loaded on SDS-PAGE. The graphs were visualized by the Image J analysis software.

### Pull-down assay

Cells were harvested and lysed in NETN buffer (50 mM Tris-HCl (pH 8.0), 150 mM NaCl, 0.5% NP-40, and 5 mM EDTA) containing protease and phosphatase inhibitors. The cell lysate was sonicated using an EpiShear™ Probe Sonicator (ACTIVE MOTIF, Carlsbad, CA, USA) and centrifuged at 13,000 rpm for 15 min at 4 °C. For pull-down of SFB-tagged proteins, the supernatant was incubated with Streptavidin-Sepharose High-Performance affinity resin (GE Healthcare) for 1 h at 4 °C and washed four times with NETN buffer. The washed precipitates were boiled with 2× sample buffer and subjected to Western blotting.

### Ubiquitination assay

Cells were treated with 10 μM of MG132 for 12 h before harvesting. Whole cells were lysed with RIPA buffer (50 mM Tris-HCl (pH 7.4), 150 mM NaCl, 1% NP-40, 0.1% SDS, 0.1% sodium deoxycholate, 5 mM EDTA, and 5 mM EGTA) containing complete protease and phosphatase inhibitors. The same amount of protein lysates (700–1000 μg) was immunoprecipitated with anti-DNA-PKcs antibody overnight at 4 °C. The next day, the immunoprecipitates were captured by incubation with Protein A Sepharose Fast-Flow (GE Healthcare) for 2 h and the beads were washed four times with RIPA buffer and boiled with 2× sample buffer for 5 min. Analysis of ubiquitination was performed by immunoblotting with anti-Ub antibody or anti-HA antibody.

### Immunofluorescence

To visualize subcellular localization, HeLa and hTERT-RPE1 cells were seeded on coverslips in 6 well plates and transfected with the indicated plasmids. At 36 h post-transfection, the cells were pretreated with 125 nM of MitoTracker-Red (Molecular Probes, Eugene, OR, USA) for 30 min before harvesting. Cells were fixed with 4% formaldehyde solution in PBS (MERCK) for 15 min at 37 °C, rinsed with PBS, and permeabilized with 0.5% Triton-X 100 in PBS for 10 min. For immunofluorescence staining, cells were blocked with 3% bovine serum albumin in PBS for 1 h at room temperature. Cells were incubated with the same buffer containing primary antibodies overnight at 4 °C, followed by incubation with secondary antibodies for 1 h at room temperature. After final washing, coverslips were mounted in VECTASHIELD mounting medium with DAPI (Vector Laboratories, Newark, CA, USA). Images were captured on a Nikon Eclipse Ti fluorescence microscope and Nikon A1R HD25_N-SIM S confocal microscope and analyzed using Nikon’s NIS-Elements Advanced Research analysis program. The mitochondrial average particle size and number per cell were quantified using ‘Object Count’ on images with pre-defined regions of interest. A complete list of the antibodies used in the study and their relative dilutions is provided in Supplementary Table 2.

### Live imaging

To observe the mitochondrial membrane potential, cells were cotransfected with mt-ro2GFP and indicated MTERF1-FokI. After 24 h, cells were stained with 50 nM Tetramethylrhodamine, methyl ester (TMRM) (Thermo Fisher Scientific, #T668) in phenol red-free DMEM containing 10% FBS at 37 °C with 5% CO_2_ for 15 min and were examined using a Zeiss LSM 800 confocal microscope with Airyscan. The fluorescence intensity of TMRM was measured by using ImageJ software. The three regions of interest were selected from the mitochondrial regions per each cell. And the fluorescence intensities of TMRM were averaged from three regions of interest per each cell.

### Quantitative RT-PCR

Total RNA from cultured cells was extracted using TRIzol reagent (Thermo Fisher Scientific) and quantified using spectrophotometer. 2 μg RNA was reverse transcribed to cDNA using PrimeScript Reverse Transcriptase (TaKaRa, Kusatsu, Japan) and real-time PCR (40 cycles) was performed using THUNDERBIRD SYBR qPCR Master Mix (TOYOBO, Osaka, Japan). Relative gene expression was normalized using human/mouse glyceraldehyde 3-phosphate dehydrogenase (GAPDH) as a housekeeping gene. A list of primers is provided in Supplementary Table 3.

### Mitochondrial DNA copy number assay

Total genomic DNA (gDNA) including mitochondrial DNA from cultured cells was extracted using Labopass Tissue Genomic DNA Isolation Kit mini (Cosmogenetech, Seoul, South Korea) and quantified by spectrophotometer. 20 ng of gDNA was subjected to real-time PCR (40 cycles) using THUNDERBIRD SYBR qPCR Master Mix (TOYOBO). Relative gene expression was normalized using human GAPDH as a housekeeping gene. A list of primers is provided in Supplementary Table 3.

### Statistical analysis

All data are presented as mean ± SD (standard deviation) of results obtained from at least three independent biological replicates. GraphPad Prism (GraphPad Software Inc, La Jolla, CA, USA) was used for statistical analysis. Statistical differences between two groups were compared by Student’s t-test. For three or more groups, statistical differences were determined by analysis of variance (ANOVA) followed by the Bonferroni multiple comparison test. *P ≤ 0.05 was considered statistically significant.

### Supplementary information

Supplementary Figure

Supplementary table 1

Supplementary table 2

Supplementary table 3

Check list

## Data Availability

All data and information concerning this study will be made available upon request.
