# Supplementary material for: Mitochondrial E3 ligase MARCH5 is a safeguard against DNA-PKcs-mediated immune signaling in mitochondria-damaged cells
Source: Cell Death Dis. 2023 Dec 1;14(12):788. doi: 10.1038/s41419-023-06315-9 (PMC10692114; doi:10.1038/s41419-023-06315-9)
Supplement: Supplementary file 1 — Supplementary Figure [file 41419_2023_6315_MOESM1_ESM.pdf]

Supplementary Fig. 1

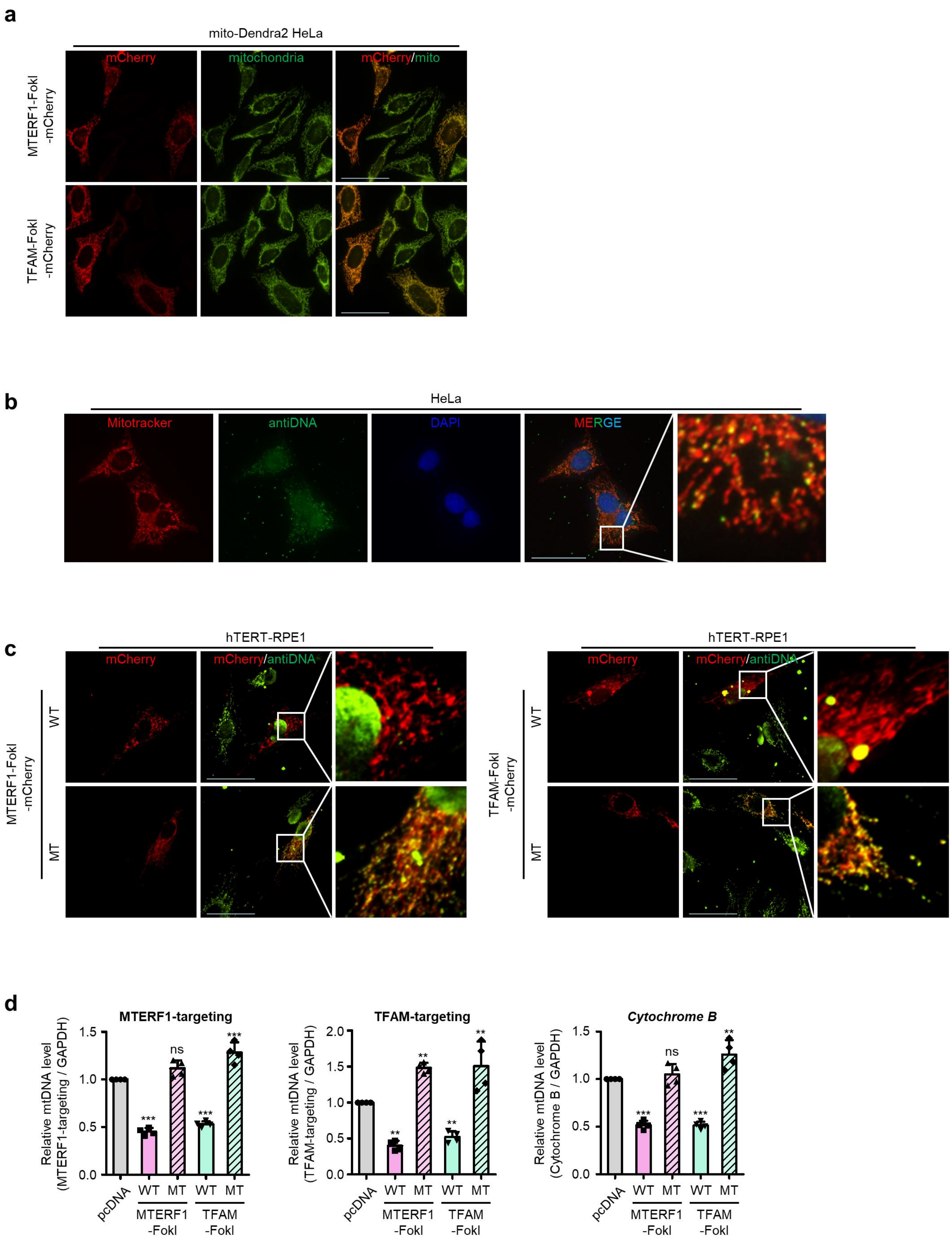

Supplementary Fig. 1 Mitochondrial DNA-targeting FokI endonuclease system to induce mtDSBs

a, Experiment as in Fig. **1c**. Representative immunofluorescence images showing the indicated mt-FokI in red and Dendra2 in green. Cells were analyzed by fluorescence microscopy 36 h after transfection with the indicated mt-FokI. Scale bar, 50 μm. **b,** Immunofluorescence analysis of mitochondrial and extranuclear dsDNA staining using Mitotracker and anti-DNA antibody in the HeLa cell line. Scale bar, 50 μm. **c,** Immunofluorescence analysis of mitochondrial DNA 36 h after transfection of hTERT-RPE1 cells with the indicated mt-FokI. Scale bar, 50 μm. **d,** qPCR for mtDNA copy number using primers derived from MTERF1-targeted, TFAM-targeted and *cytochrome B* sequences in the same sample shown in Fig. **1f**. Normalized expression Data are means ± SD of $n = 4$ and the normalized error are shown. Each experiment was performed at least three times. $^{**}P \leq 0.01$, $^{***}P \leq 0.005$, ns, not significant.

Supplementary Fig. 2

a

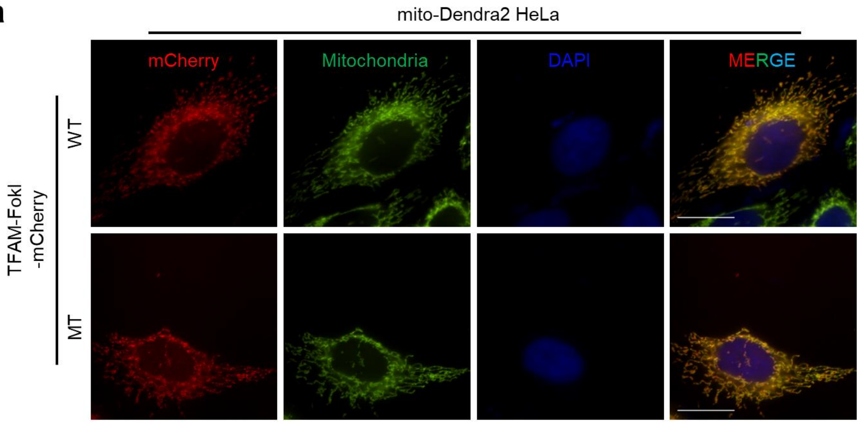

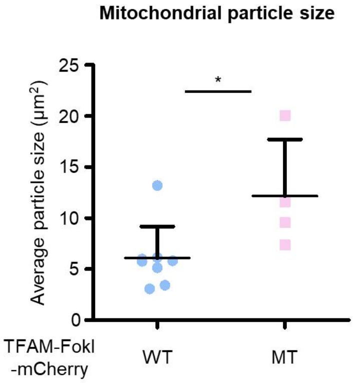

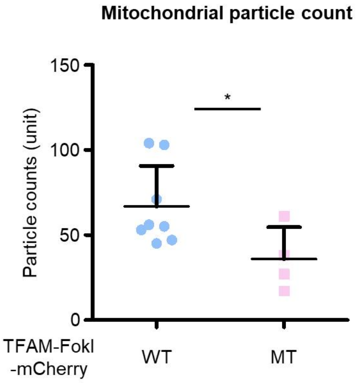

b

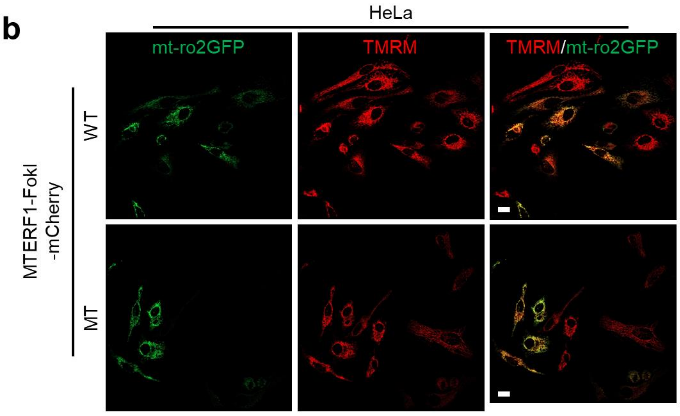

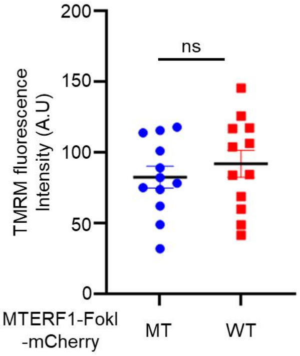

Supplementary Fig. 2 Mitochondrial parameters following expression of the mitochondrial DNA-targeting FokI

a, Representative images. Mito-Dendra2 HeLa cells were transfected with the indicated TFAM-FokI for 36h. Scale bar, 25 μm. Scatter plots were visualized using Nikon's NIS-Elements Advanced Research analysis program. Mitochondrial morphology was quantified by measuring the mean particle area and particle number. The graph was presented as mean ± SD. Unpaired two-tailed t-test was used, *n* = 8, 4. **b,** Representative images of TMRM staining after cotransfection with the indicated MTERF1-FokI and mt-ro2GFP for 24h in HeLa cells. Scale bar, 20 μm. Fluorescence intensity of TMRM was measured by using ImageJ software. The mitochondrial membrane potential was measured in four cells from each of the three images. The graph was shown as mean ± SEM. *n* =12. Each experiment was performed at least three times. *$P \leq 0.05$, ns, not significant.

Supplementary Fig. 3

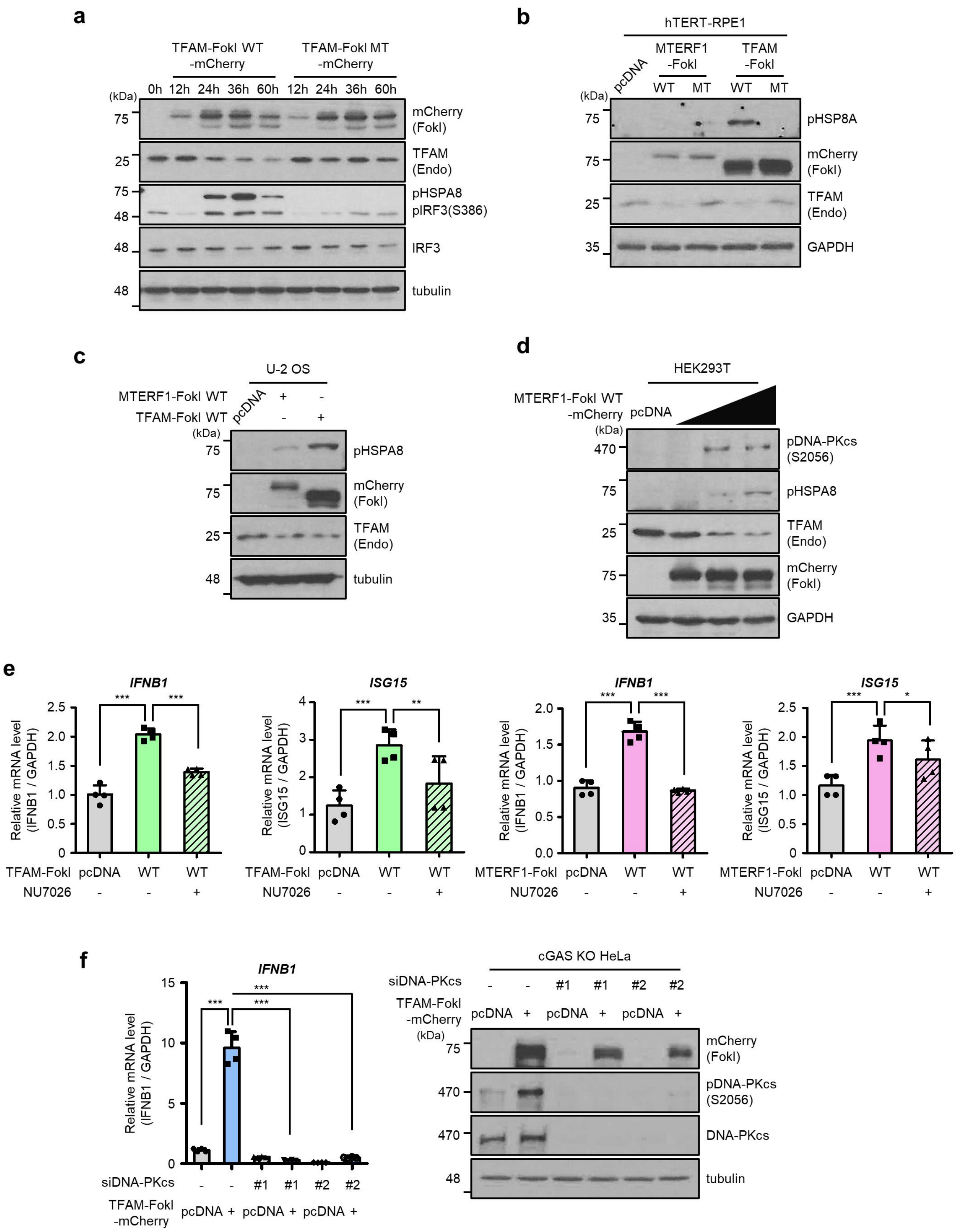

Supplementary Fig. 3 DNA-PKcs-mediated type I IFN response by mtDSBs in various cell lines

a, Western blot to analyze the levels of pIRF3 and pHSPA8 in HEK293T harvested after expression of TFAM-FokI WT or MT for the indicated times. **b, c,** Western blot analysis for pHSPA8 levels in hTERT-RPE1 cells and U-2 OS cells collected 36 h after transfection with the indicated mt-FokI. **d,** Western blot analysis to confirm the protein of pDNA-PKcs and pHSPA8 in HEK293T cells transfected with MTERF1-FokI WT in a dose-dependent manner. **e,** Analysis of *IFNB1*, *ISG15* mRNA levels in *cGAS* KO HeLa cells expressing TFAM-FokI WT or MTERF1-FokI WT pretreated with or without NU7026. Normalized expression Data are means ± SD of $n = 4$; one-way ANOVA with CI = 95% and Bonferroni's post hoc test. **f,** Left, RT-qPCR to analyze *IFNB1* mRNA levels in *cGAS* KO HeLa cells expressed TFAM-FokI WT for 36 h after DNA-PKcs knockdown induction. Right, Western blot analysis of DNA-PKcs in the same samples. Data are means ± SD of $n = 4$; one-way ANOVA with CI = 95%. Each experiment was performed at least three times. *$P \leq 0.05$, **$P \leq 0.01$, ***$P \leq 0.005$.

Supplementary Fig. 4

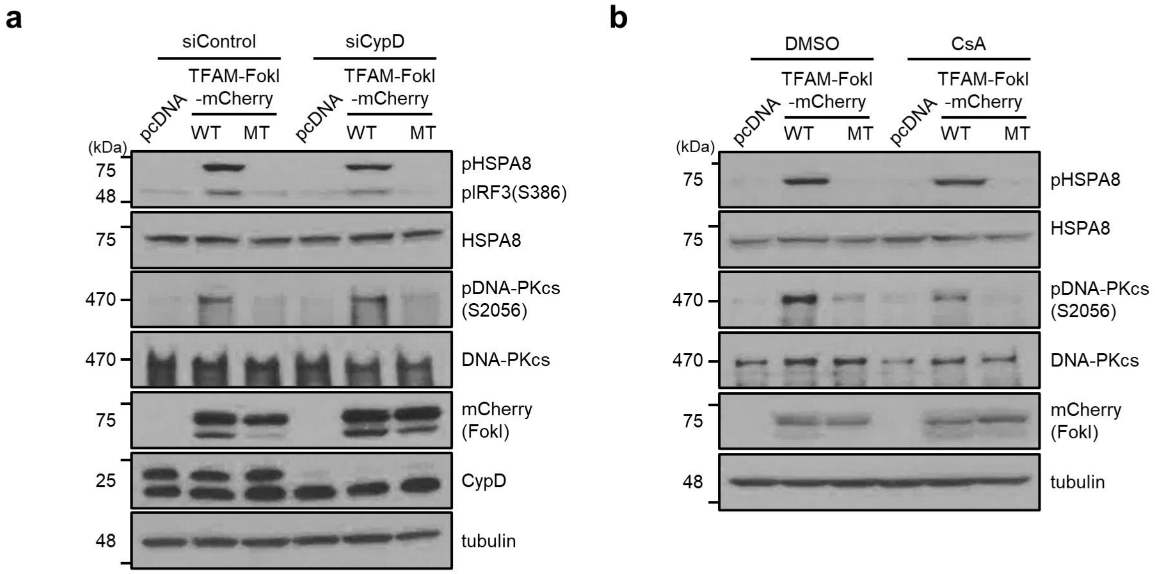

Supplementary Fig. 4 Inhibition of mPTP did not significantly affect the activation of DNA-PKcs and HSPA8.

a, Western blot to analyze the levels of pHSPA8, pIRF3 and pDNA-PKcs after transfection of the indicated TFAM-FokI for 36h in HEK293T cells transfected with siCypD. **b,** Western blot analysis of pHSPA8 and pDNA-PKcs in cells collected 36 h after expression of the indicated TFAM-FokI in HEK293T cells treated with 10 μg/ml CsA 24 h before harvest. Each experiment was performed at least three times.

Supplementary Fig. 5

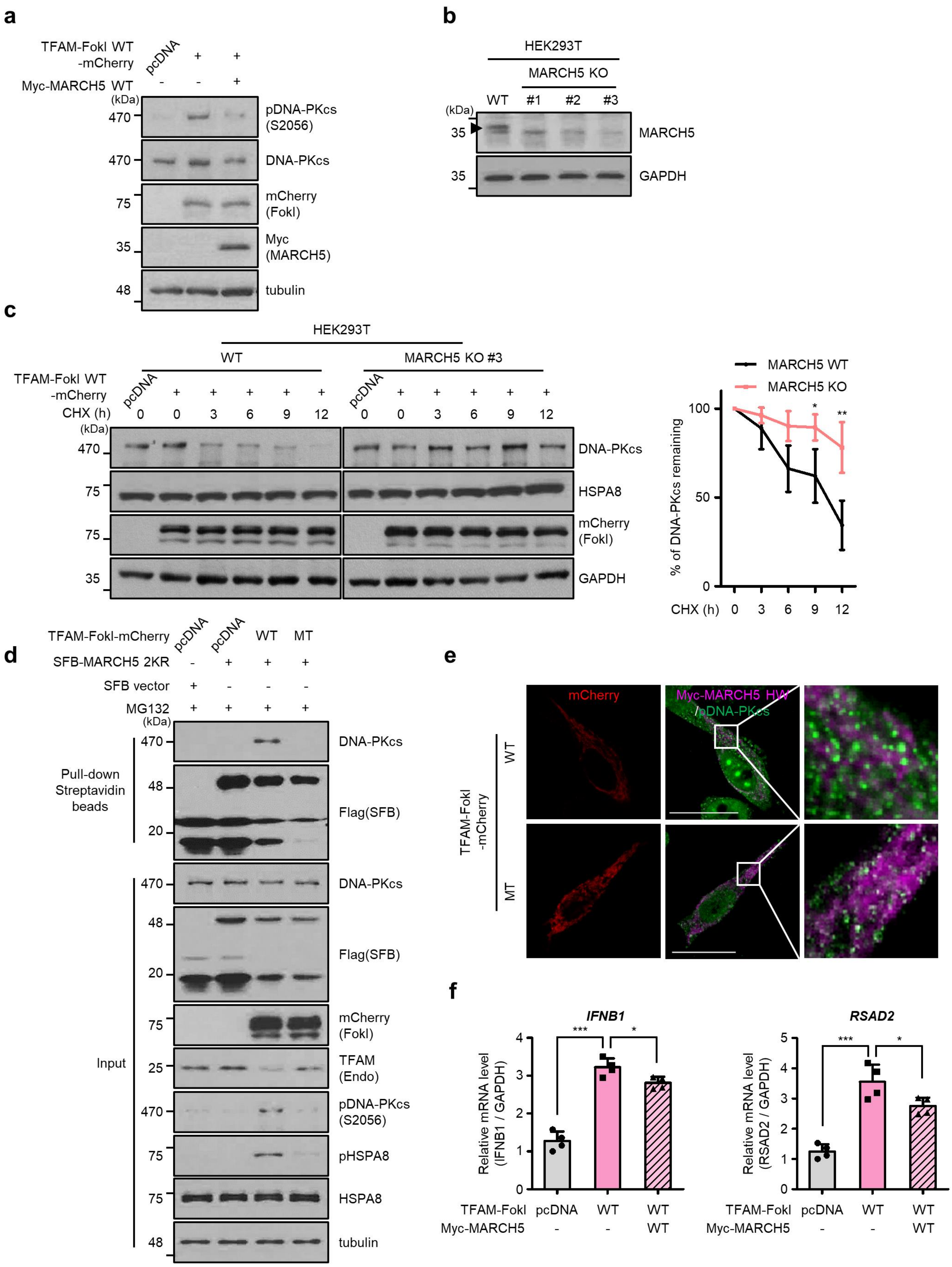

Supplementary Fig. 5 MARCH5 regulates DNA-PKcs-mediated type I interferon response

a, Western blot analysis of pDNA-PKcs and DNA-PKcs in HEK293T cells cotransfected TFAM-FokI WT with MARCH5 WT for 36 h. **b,** Western blot analysis to confirm endogenous MARCH5 in WT and MARCH5 KO HEK293T cells. **c,** Western blot analysis of DNA-PKcs and HSPA8 levels in WT and MARCH5 KO HEK293T cells transfected with TFAM-FokI WT and treated with 15 μg/ml CHX for the indicated times. Graph, Normalized expression Data are means ± SD of *n* = 3 independent experiments; two-way ANOVA with CI = 95% and Bonferroni's post hoc test. **d,** Analysis of the interaction between DNA-PKcs and MARCH5 in HEK293T cells. Cells were cotransfected with the indicated TFAM-FokI and SFB-MARCH5 2KR and harvested 36 h later. Cells were treated with 10 μM MG132 12 h before harvest. Cell lysates were immunoprecipitated with streptavidin beads and then immunoblotted with the antibodies against indicated proteins. **e,** Immunofluorescence staining using a pDNA-PKcs and Myc antibody after transfection of the indicated TFAM-FokI into cGAS KO HeLa cells overexpressing Myc-MARCH5 HW. Representative images taken by confocal microscopy with MARCH5 HW labelled in pink, pDNA-PKcs in green, and TFAM-FokI in red. Scale bar, 25 μm. **f,** RT-qPCR analysis of *IFNB1* and *RSAD2*, 36 h after cotransfection of TFAM-FokI WT and MARCH5 WT in *cGAS* KO HeLa cells. Data are means ± SD of *n* = 4; one-way ANOVA with CI = 95% and Bonferroni's post hoc test. Each experiment was performed at least three times. $^*P \leq 0.05$, $^{**}P \leq 0.01$, $^{***}P \leq 0.005$.

Supplementary Fig. 6

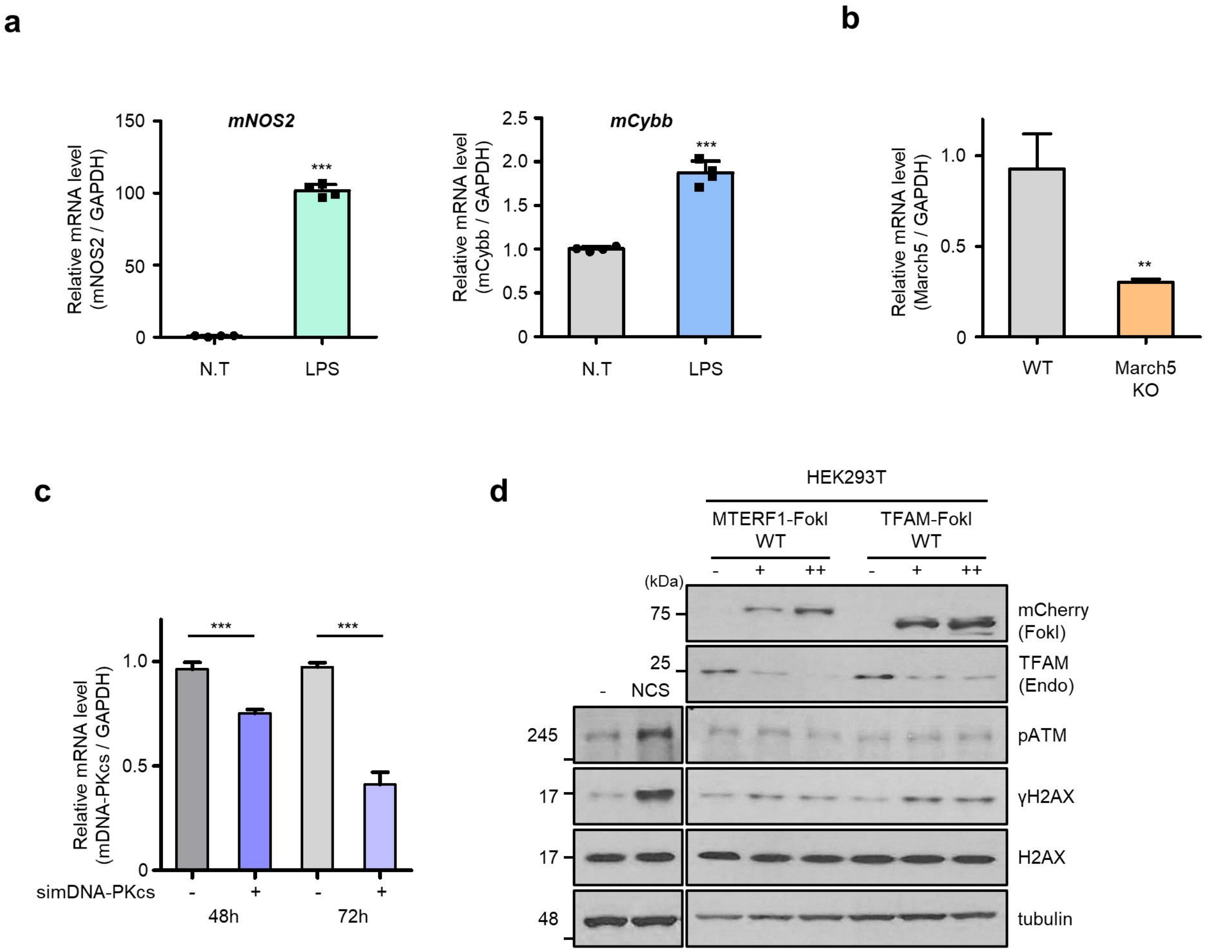

Supplementary Fig. 6 Production of NO and ROS by LPS treatment and gene depletion in murine macrophages

a, RT-qPCR analysis of *mNOS2* and *mCybb* mRNA levels using the same sample as in Fig. **6a**. Data are means ± SD of $n = 4$; one-tailed ratio unpaired Student's t-test with CI = 95%. **b,** RT-qPCR analysis of *March5* mRNA levels in WT and *March5* KO RAW 264.7 cells. Data are means ± SD. **c,** RT-qPCR analysis of *mDNA-PKcs* mRNA levels in *March5* KO RAW 264.7 cells transfected with siRNA targeting *mDNA-PKcs* for the indicated times. Data are presented mean ± SD. **d,** Western blot analysis of pATM and γH2AX in HEK293T cells collected 36 h after transfection with the indicated MTERF1- and TFAM-FokI. Treatment with 100 ng/ml NCS for 4 h was used as a positive control. Each experiment was performed at least three times. **$P \leq 0.01$, ***$P \leq 0.005$.
